# Effects of dietary *ε*-polylysine supplementation on growth performance, antioxidant capacity, immune function, and intestinal microbiota in growing male minks

**DOI:** 10.3389/fmicb.2025.1569620

**Published:** 2025-05-13

**Authors:** Nianxue Wang, Heliang Wang, Xiaowen Zhang, Lianwen Zhao, Wenli Li, Beibei Zhang

**Affiliations:** ^1^College of Animal Science and Technology, Qingdao Agricultural University, Qingdao, China; ^2^Animal Health Development Research Center, Qingdao Qiushi College, Qingdao, China

**Keywords:** ε-polylysine, mink, growth performance, antioxidant capacity, immune function, gut microbiota

## Abstract

**Introduction:**

This study investigated the effects of dietary *ε*-polylysine (ε-PL) supplementation on the growth performance, antioxidant capacity, immune function and intestinal microbiota in growing male minks.

**Methods:**

Ninety-six 12-week-old male minks were randomly divided into 6 treatments (8 replicates per treatment and 2 minks per replicate). Minks were fed basal diets supplemented with 0 (control), 100, 200, 300, 400, or 500 mg/kg *ε*-PL for 8 weeks.

**Results:**

Compared with the control, 300–500 mg/kg ε-PL significantly increased the average daily gain (*p* < 0.05), and significantly decreased the feed-to-gain ratio (*p* < 0.05) during the whole period and significantly enhanced the body weight at week 8 (*p* < 0.05), 300–400 mg/kg *ε*-PL significantly increased the fresh pelt weight (*p* < 0.05). Compared with the control, 300 mg/kg *ε*-PL significantly increased serum T-SOD activity and jejunal mucosal T-SOD and GSH-Px activities (*p* < 0.05). Compared to the control, 200–400 mg/kg *ε*-PL significantly increased serum IgA level (*p* < 0.05), 300–400 mg/kg *ε*-PL significantly increased serum IgM level (*p* < 0.05), 400–500 mg/kg *ε*-PL significantly increased serum IgG level (*p* < 0.05). Compared with the control, 200–400 mg/kg *ε*-PL significantly increased jejunal mucosal IgA level (*p* < 0.05), 100 mg/kg ε-PL significantly increased jejunal mucosal IgM level (*p* < 0.05), 100–400 mg/kg *ε*-PL significantly increased jejunal mucosal IgG level (*p* < 0.05). Compared with the control group, all ε-PL supplemented groups significantly decreased serum IL-2 and IL-8 levels compared to the control (*p* < 0.05). The 16S rRNA sequencing analysis revealed that compared to the control, 300 mg/kg *ε*-PL significantly increased the relative abundance of Firmicutes and *Clostridium_sensu_stricto_1* (*p* < 0.05), and significantly decreased the relative abundance of Proteobacteria and *Escherichia-Shigella* in ileal mucosa (*p* < 0.05). Spearman correlation analysis indicated that the relative abundance of *Escherichia-Shigella* was negatively correlated with the growth performance. The relative abundance of *Clostridium_sensu_stricto_1* was positively correlated with the jejunal mucosal antioxidant indicators and immunoglobulin levels.

**Discussion:**

In conclusion, dietary *ε*-PL supplementation can improve growth performance, antioxidant capacity, immune function, and gut microbial community in growing male minks, and the optimal dosage of *ε*-PL is 300 mg/kg.

## 1 Introduction

The mink (*Mustela vison*) is a carnivorous mammal belonging to the Mustelidae family. Its fine and soft fur serves as premium raw material for making fur coats, hats, and other luxury products. Mink diets are wet-type feed primarily formulated with fresh or frozen ingredients including fish, fish by-products, eggs, and poultry by-products. The growing stage of minks occurs during the hot summer, when feed is prone to spoilage and deterioration, increasing the risk of diarrhea and other diseases. Furthermore, high temperatures also easily cause heat stress reactions in animals, leading to decreased antioxidant function, elevated inflammatory response, impaired intestinal health, and disruption of gut microbiota, which adversely affects overall health (Cao et al., 2021; Ortega and Szabó, 2021). Given these risks, it is crucial to explore effective additives that can maintain feed quality and protect health of the minks. One promising candidate is *ε*-Polylysine (ε-PL), a natural antimicrobial peptide that offers multiple benefits for animal health and nutrition.

*ε*-PL is a polypeptide consisting of 25–35 lysine residues, produced by the fermentation of *Streptomyces albus* (Huang et al., 2025). It is known that *ε*-PL has broad-spectrum antimicrobial activity in neutral and slightly acidic conditions (Yoshida and Nagasawa, 2003). Since *ε*-PL is a positively charged peptide, it can readily bind to the sites with negative charges on the surface of bacterial and fungal cells, disrupt cell surface structures, interfere with cellular metabolism, and inhibit their growth and reproduction (Deng et al., 2020). *ε*-PL has been broadly used as an antiseptic and bacteriostatic agent in food and pharmaceutical fields (Alemán et al., 2016; Yue et al., 2022; Sun et al., 2019). In addition, *ε*-PL can be partially degraded into L-lysine, thereby supplementing essential amino acids for the organism (Hiraki et al., 2003). The use of *ε*-PL as a feed additive in the livestock industry has been reported in a limited number of studies. Recent studies have reported that dietary ε-PL supplementation improves egg-laying performance and immune function, reduces oxidative damage, increases intestinal microbiota diversity, and maintains intestinal health in laying hens (Wang et al., 2024). Dietary supplementation of *ε*-PL affects nutrient and energy utilization and regulates the structure of gut microbiota structure in Ningxiang pigs (Zhang et al., 2020a). Furthermore, dietary *ε*-PL addition increases jejunal villus height and reduces crypt depth in mice, improving the intestinal morphology (Zhang et al., 2022).

However, the effects of ε-PL on the growth, antioxidant and immune function, and gut microbiota in minks remains unclear. The aim of this study was to assess the effects of dietary ε-PL supplementation on growth performance, antioxidant capacity, immune function, and intestinal microbiota in growing male minks.

## 2 Materials and methods

### 2.1 Animals and experimental design

The experimental procedure was approved by the Animal Care and Use Committee of Qingdao Agricultural University (No. DKY20230526). A total of 96 red-eyed white male minks (aged 12 weeks) with similar body weights were randomly divided into six groups, with 8 replicates (cages) per group and 2 minks per replicate. The minks were fed basal diets supplemented with 0 (control), 100, 200, 300, 400, or 500 mg/kg *ε*-PL. The trial lasted for 8 weeks following a week of adaption. All minks were housed in standard metal cages (L 75 cm × W 30 cm × H 45 cm) aligned in two parallel rows within a naturally ventilated shed structure. They were fed at 5:00 and 17:00 every day with free access to water. Table 1 shows the composition and nutrient level of the basal diet. ε-PL was supplemented in the form of ε-polylysine hydrochloride (99% purity, Zhengzhou Bainafo Bioengineering, Zhengzhou, Shangdong, China). All minks had completed canine distemper and parvovirus vaccinations.

**Table 1:** Composition and nutrient levels of basal diets (air-dry basis, %).

Items	Weeks 0–4	Weeks 5–8
Sea fishes	13	8
Unhatched fertilized egg	32	32
Chicken head	20	20
Extruded corn	10	10
Monkfish head	14	18
Lepidotrigla	5	6
Lard	1	2
Soybean meal	2	2
Premix^a^	3	2
Total	100	100
Nutrient levels		
ME (MJ/kg)^b^	15.98	17.04
Ether extract^c^	16.65	19.85
Crude protein^c^	31.81	31.26
Calcium^c^	2.47	2.59
Phosphorus^c^	1.59	1.64

a The premix contains the following nutrients (per kg of the diets): VA 9,000 IU, VC 40 mg, VE 20 mg, VK_3_ 0.5 mg, VB_1_ 5 mg, VB_2_ 3 mg, VB_6_ 2.5 mg, VB_12_ 1 mg, VD_3_ 2,000 IU, nicotinic acid 20 mg, pantothenic acid 6 mg, folic acid 0.5 mg, biotin 0.5 mg, Fe 30 mg, Zn 25 mg, Mn 10 mg, Cu 5 mg, I 0.25 mg, and Se 0.2 mg.

b Calculated value.

c Measured value.

### 2.2 Growth performance measurement

Body weights of minks were measured at the start of the trial (week 0) and at the end of week 4 and week 8. The feed intake was monitored for 3 consecutive days each week. The average daily gain (ADG), average daily feed intake (ADFI), and the feed-to-gain ratio (F/G) were calculated. At the end of the trial, one mink was randomly chosen from each replicate, and the body length was measured from the tip of the nose to the base of the tail. After the minks were sacrificed by injecting air into the heart, the pelt was then removed and the fresh pelt weight was determined. The pelt length was measured as the distance from the nose tip to the tail base of the pelt.

### 2.3 Sample collection

From each selected mink, 10 mL of blood was drawn from the heart and subjected to centrifugation at 3,000 r/min for 10 min at 4°C to separate the serum. The obtained serum was subsequently stored at −20°C. After slaughtering, the jejunum and ileum were resected, cut open, and rinsed with saline. The jejunal and ileal mucosa were collected, snap-frozen, and stored at −80°C.

### 2.4 Determination of antioxidant indicators, immunoglobulin (Ig) levels, and cytokine levels

The activities of total antioxidant capacity (T-AOC; a015-1-2), total superoxide dismutase (T-SOD; a001-1-2), and glutathione peroxidase (GSH-Px; a005-1-2), as well as the level of malondialdehyde (MDA; a003-1-2) in serum and jejunal mucosa were measured using the commercial assay kits (Nanjing Jianjian Bioengineering Research Institute, Nanjing, China). The levels of IgG (H106-1-2), IgA (H108-1-2), and IgM (H109-1-2) in serum and jejunal mucosa, as well as interleukin (IL)-1β (H002-1-2), IL-2 (H003-1-2), IL-8 (H008-1-2), and IL-10 (H009-1-2) in serum, were measured using the corresponding enzyme-linked immunosorbent assay (ELISA) kits (Nanjing Jianjian Bioengineering Research Institute, Nanjing, China). Jejunal mucosa was homogenized in 0.9% sodium chloride to prepare a 10% mucosal homogenate, which was centrifuged at 3000 rpm for 10 min at 4°C to collect the supernatant. Protein levels in jejunal mucosal homogenate were measured using BCA assay kits (CW0014S; Cwbio, Beijing, China). Jejunal antioxidant indicators and immunoglobulin levels were expressed as units per milligram of protein.

### 2.5 Sequencing of the gut microbiota

Based on the results of growth performance, antioxidant capacity and immune function, 300 mg/kg *ε*-PL was identified as the optimal supplementation level. Therefore, minks fed 0 and 300 mg/kg ε-PL were selected for sequencing the gut microbiota. Briefly, total genomic DNA was extracted from the ileal mucosa of minks using a Mag-bind soil DNA kit (M5635-02; Omega Biotek, GA, USA), and the purity and concentration of DNA were measured. The V3-V4 variable region was amplified using PCR with the primers 341F (5’-CCTAYGGGRBGCASCAG-3′) and 806R (5’-GGACTACNNGGGTATCTAAT-3′). After isolation using 2% agarose gel electrophoresis, target fragments were cut from the gel, recovered using a Quant-iT PicoGreen dsDNA assay kit (P7589; Invitrogen, Carlsbad, CA, USA) and quantified on an FLx800 microplate reader (BioTek, USA). After construction using a TruSeq Nano DNA LT Library Prep kit (20,015,965; Illumina, USA), the library was analyzed using Agilent Bioanalyzer 2,100 and Promega QuantiFluor. After qualification, the library was sequenced using the NovaSeq 6,000 system. The 16S rRNA sequencing analysis was conducted at Applied Protein Technology (APTBIO, Shanghai, China). Sequences with more than 97% similarity were set as a taxonomic operating unit. Qiime2 was used to calculate the *α* and *β* diversity indices, and LEfSe was utilized to analyze the significant differences at each taxonomic level.

### 2.6 Statistical analysis

Data were analyzed using a one-way ANOVA procedure in SPSS 25.0 software (SPSS Inc., Chicago, IL, USA). Differences between treatments were assessed by Duncan’s multiple-range tests. The effects of dietary *ε*-PL supplementation levels were evaluated using linear and quadratic polynomial contrasts. The sequencing data on the gut microbiota from the control and 300 mg/kg groups were subjected to an independent-sample t-test. Spearman’s correlation analysis was used to assess the correlation between gut microbiota and health-related indicators. Data are expressed as means and pooled SEM. Differences were considered statistically significant at *p* < 0.05.

## 3 Results

### 3.1 Effects of *ε*-PL on growth performance

As presented in Table 2, the body weight at week 4 increased in a linear manner (*p* < 0.05). Compared to the control, 200, 300, 400 and 500 mg/kg *ε*-PL significantly increased the body weight at week 4 (*p* < 0.05). The linear and quadratic effects on the body weight were observed at week 8 when *ε*-PL levels increased (*p* < 0.05). Compared to the control, 300, 400 and 500 mg/kg *ε*-PL significantly increased the body weight at week 8 (*p* < 0.05). Increasing levels of *ε*-PL linearly increased the ADG during weeks 0–4 and 5–8, and both linearly and quadratically increased the ADG during weeks 0–8 (*p* < 0.05). Compared to the control group, 300, 400, and 500 mg/kg *ε*-PL significantly increased the ADG during weeks 0–4 and 0–8 (*p* < 0.05). Additionally, 300 and 400 mg/kg ε-PL significantly increased the ADG during weeks 5–8 (*p* < 0.05). The F/G was significantly decreased in a linear and quadratic manner as *ε*-PL levels increased during weeks 0–4 and 5–8 (*p* < 0.05). Moreover, ε-PL supplementation linearly decreased the F/G during weeks 0–8 (*p* < 0.05). Compared to the control, 200, 300, 400, and 500 mg/kg *ε*-PL significantly decreased the F/G during weeks 0–4 (*p* < 0.05). Furthermore, 300, 400, and 500 mg/kg *ε*-PL significantly reduced the F/G during weeks 5–8 and 0–8 (*p* < 0.05). Fresh pelt weight at week 8 increased linearly and quadratically as *ε*-PL levels increased (*p* < 0.05). Compared to the control, 300 and 400 mg/kg *ε*-PL significantly increased the fresh pelt weight at week 8 (*p* < 0.05). No significance was observed in the body length and fresh pelt length at week 8 (*p* > 0.05).

**Table 2:** Effects of dietary ε-PL supplementation on growth performance of growing male minks (*n* = 8).

Items	ε-PL levels (mg/kg)						SEM	*p* value		
	0	100	200	300	400	500		ANOVA	Linear	Quadratic
Body weight (g)										
Week 0	1284.38	1279.38	1267.5	1,290	1293.13	1283.13	6.211	0.894	0.666	0.810
Week 4	1678.00^b^	1762.00^ab^	1804.00^a^	1810.71^a^	1815.71^a^	1826.00^a^	14.326	0.025	0.002	0.086
Week 8	2041.86^b^	2210.00^ab^	2233.57^ab^	2402.86^a^	2377.86^a^	2308.57^a^	33.838	0.011	0.002	0.044
ADG (g)										
Weeks 0–4	15.11^c^	16.07^bc^	17.18^abc^	19.02^a^	17.74^ab^	17.83^ab^	0.366	0.024	0.004	0.066
Weeks 5–8	14.46^c^	16.48^bc^	17.20^abc^	19.79^ab^	21.66^a^	17.92^abc^	0.658	0.020	0.005	0.074
Weeks 0–8	13.53^b^	16.77^ab^	16.74^ab^	19.76^a^	18.24^a^	17.79^a^	0.574	0.039	0.011	0.045
ADFI (g)										
Weeks 0–4	261.28^ab^	267.04^ab^	254.15^b^	267.49^ab^	274.58^a^	264.71^ab^	2.010	0.042	0.165	0.966
Weeks 5–8	290.82	301.04	294.58	315.93	310.29	306.63	4.133	0.503	0.140	0.483
Weeks 0–8	275.95	276.69	275.46	288.8	289.22	282.7	3.291	0.698	0.226	0.696
F/G										
Weeks 0–4	18.92^a^	17.27^ab^	15.16^bc^	13.85^c^	15.08^bc^	14.82^bc^	0.449	0.008	0.001	0.020
Weeks 5–8	21.03^a^	18.96^ab^	17.99^abc^	15.62^c^	14.76^c^	17.59^bc^	0.510	0.002	0.001	0.012
Weeks 0–8	20.31^a^	17.60^ab^	17.17^ab^	15.02^b^	16.50^b^	16.13^b^	0.476	0.027	0.005	0.051
Body length (cm)	46.50	47.88	47.63	47.25	46.50	46.25	0.266	0.385	0.296	0.096
Fresh pelt length (cm)	57.88	59.50	57.75	58.50	58.13	55.88	0.477	0.405	0.177	0.201
Fresh pelt weight (g)	620.00^c^	687.50^c^	725.00^bc^	845.00^ab^	884.17^a^	757.86^abc^	23.816	0.005	0.001	0.036

ADG, average daily gain; ADFI, average daily feed intake; F/G, the ratio of feed to gain. Means in the same row with different superscripts are significantly different (*p* < 0.05).

### 3.2 Effects of *ε*-PL on the antioxidant function of serum and jejunal mucosa

As shown in Table 3, increasing *ε*-PL levels quadratically enhanced serum T-AOC level (*p* < 0.05), and both linearly and quadratically elevated T-SOD activity (*p* < 0.05). Serum T-AOC level was significantly higher in the 200 and 300 mg/kg *ε*-PL groups than in the 500 mg/kg group (*p* < 0.05). Compared to the control, 300 mg/kg *ε*-PL significantly increased serum T-SOD activity (*p* < 0.05).

**Table 3:** Effects of dietary ε-PL supplementation on the antioxidant activities in serum and jejunal mucosa of growing male minks (*n* = 8).

Items	ε-PL levels (mg/kg)						SEM	*p* value		
	0	100	200	300	400	500		ANOVA	Linear	Quadratic
Serum										
T-AOC (U/mL)	8.86^ab^	8.99^ab^	10.04^a^	9.91^a^	8.77^ab^	8.47^b^	0.178	0.034	0.444	0.006
T-SOD (U/mL)	253.69^b^	256.27^b^	269.68^b^	309.07^a^	290.76^ab^	272.90^b^	5.472	0.028	0.021	0.039
GSH-Px (U/mL)	1340.71	1339.89	1359.18	1428.31	1345.47	1287.72	16.684	0.295	0.598	0.067
MDA (nmol/mL)	13.99	13.77	14.00	12.58	13.15	12.35	0.297	0.435	0.066	0.821
Jejunal mucosa										
T-AOC (U/mg prot)	2.27	2.48	2.41	2.43	2.18	2.09	0.071	0.562	0.245	0.179
T-SOD (U/mg prot)	79.31^b^	78.01^b^	82.41^ab^	89.81^a^	78.74^b^	75.82^b^	1.379	0.044	0.770	0.020
GSH-Px (U/mg prot)	29.01^b^	33.79^ab^	33.22^ab^	37.52^a^	30.68^b^	29.27^b^	0.852	0.033	0.825	0.004
MDA (nmol/mg prot)	1.62	1.52	1.94	1.46	1.72	1.80	0.082	0.572	0.541	0.899

T-AOC, total antioxidant capacity; T-SOD, total superoxide dismutase; GSH-Px, glutathione peroxidase; MDA, malondialdehyde. Means in the same row with different superscripts are significantly different (*p* < 0.05).

The jejunal mucosal T-SOD and GSH-Px activities showed a quadratic effect with increasing *ε*-PL levels (*p* < 0.05). The supplementation of 300 mg/kg *ε*-PL significantly increased the activities of T-SOD and GSH-Px in jejunal mucosa relative to the control (*p* < 0.05).

### 3.3 Effects of *ε*-PL on serum and jejunal mucosal immunoglobulin levels

As listed in Table 4, increasing levels of *ε*-PL elevated serum levels of IgA, IgM, and IgG in a linear manner (*p* < 0.05). Compared to the control, 200, 300 and 400 mg/kg *ε*-PL significantly increased serum IgA level (*p* < 0.05). Compared with the control group, 300 and 400 mg/kg *ε*-PL supplementation significantly increased serum IgM level (*p* < 0.05). Serum IgG level was significantly enhanced by 400 and 500 mg/kg *ε*-PL relative to the control (*p* < 0.05).

**Table 4:** Effects of dietary ε-PL supplementation on the immunoglobulin levels in serum and jejunal mucosa of growing male minks (*n* = 8).

Items	ε-PL levels (mg/kg)						SEM	*p* value		
	0	100	200	300	400	500		ANOVA	Linear	Quadratic
Serum										
IgA (g/L)	0.25^c^	0.25^c^	0.26^b^	0.27^ab^	0.27^a^	0.26^abc^	0.002	0.016	0.017	0.085
IgM (g/L)	1.96^b^	1.94^b^	1.96^b^	2.18^a^	2.13^a^	2.10^ab^	0.026	0.005	0.002	0.473
IgG (g/L)	28.48^c^	28.18^c^	28.96^bc^	30.12^abc^	31.89^a^	30.97^ab^	0.371	0.011	0.001	0.908
Jejunal mucosa										
IgA (μg/mgprot)	39.46^b^	45.76^ab^	52.33^a^	48.23^a^	46.60^a^	45.54^ab^	1.035	0.011	0.136	0.002
IgM (μg/mgprot)	372.23^b^	531.11^a^	440.65^ab^	450.78^ab^	450.51^ab^	448.75^ab^	13.494	0.017	0.529	0.106
IgG (mg/mgprot)	4.28^c^	5.30^ab^	5.31^ab^	5.61^a^	5.00^ab^	4.89^bc^	0.108	0.004	0.194	<0.001

IgA, immunoglobulin A; IgM, immunoglobulin M; IgG, immunoglobulin G. Means in the same row with different superscripts are significantly different (*p* < 0.05).

The IgA and IgG levels in jejunal mucosa increased quadratically with increasing *ε*-PL levels (*p* < 0.05). Compared to the control, 200, 300 and 400 mg/kg ε-PL significantly increased jejunal mucosal IgA level (*p* < 0.05). Additionally, 100, 200, 300 and 400 mg/kg *ε*-PL significantly enhanced the IgG level in jejunal mucosa (*p* < 0.05). When compared with the control group, only 100 mg/kg ε-PL group significantly enhanced IgM level (*p* < 0.05).

### 3.4 Effects of ε-PL on serum levels of inflammatory cytokines

As presented in Table 5, serum levels of IL-2 and IL-8 decreased linearly as *ε*-PL levels increased (*p* < 0.05). When compared to the control group, 100, 200, 300, 400 and 500 mg/kg ε-PL significantly decreased serum levels of IL-2 and IL-8 (*p* < 0.05).

**Table 5:** Effects of dietary ε-PL supplementation on serum inflammatory cytokine levels in growing male minks (*n* = 8).

Items	ε-PL levels (mg/kg)						SEM	*P* value		
	0	100	200	300	400	500		ANOVA	Linear	Quadratic
IL-1β (pg/mL)	163.53	157.93	155.80	164.21	151.93	154.87	1.946	0.416	0.188	0.961
IL-2 (pg/mL)	274.43^a^	240.39^b^	233.88^b^	246.13^b^	239.90^b^	236.70^b^	3.849	0.043	0.021	0.062
IL-8 (pg/mL)	109.97^a^	89.20^b^	92.04^b^	98.46^b^	89.71^b^	92.05^b^	1.832	0.009	0.018	0.067
IL-10 (pg/mL)	54.27	50.94	51.78	54.60	49.60	50.37	0.721	0.231	0.154	0.855

IL-1β, interleukin-1β; IL-2, interleukin-2; IL-8, interleukin-8; IL-10, interleukin-10. Means in the same row with different superscripts are significantly different (*p* < 0.05).

### 3.5 Effects of *ε*-PL on the composition of intestinal microbiota

#### 3.5.1 Alpha diversity and beta diversity

**Figure 1:**
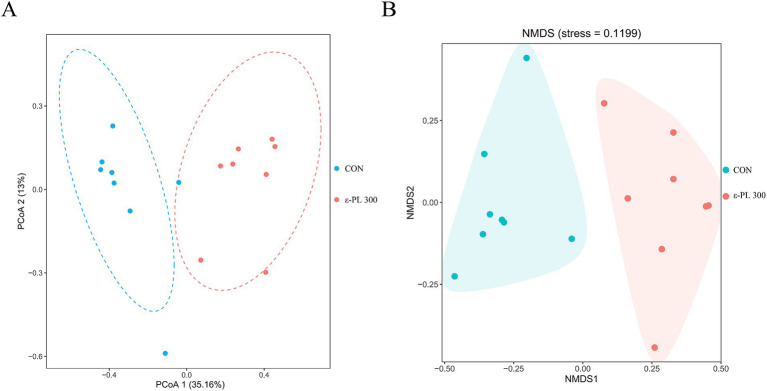
Effects of ε-PL supplementation on the beta diversity of intestinal microbiota in growing minks (*n* = 8). **(A)** Principal coordinates analysis (PCoA) based on Bray-Curtis distance; **(B)** Non-metric multidimensional scaling (NMDS) based on Bray-Curtis distance. CON, minks fed a basal diet; ε-PL 300, minks fed a basal diet supplemented with 300 mg/kg of ε-PL.

Table 6 shows that 300 mg/kg ε-PL had no significant effects on the Ace, Chao1, Shannon, Simpson, and Coverage index compared to the control (*p* > 0.05). However, PCoA and NMDS analyses revealed that the structure of the intestinal microbiota of control minks could be clearly distinguished from that of minks fed with 300 mg/kg *ε*-PL (Figures 1A,B), suggesting that dietary supplementation of 300 mg/kg ε-PL remarkably changed the intestinal microbiota in minks.

**Table 6:** Effects of dietary ε-PL supplementation on the alpha diversity indices of intestinal microbiota in growing male minks (*n* = 8).

Indices	ε-PL levels (mg/kg)		SEM	*P* value
	0	300		
Observed_species	113.13	85.13	19.767	0.498
Ace index	122.88	96.74	20.750	0.547
Chao1 index	119.16	93.54	20.472	0.550
Shannon index	3.30	2.80	0.302	0.432
Simpson index	0.81	0.68	0.041	0.092
Coverage index	0.9993	0.9992	<0.001	0.670

#### 3.5.2 Relative abundance of bacterial taxa

**Figure 2:**
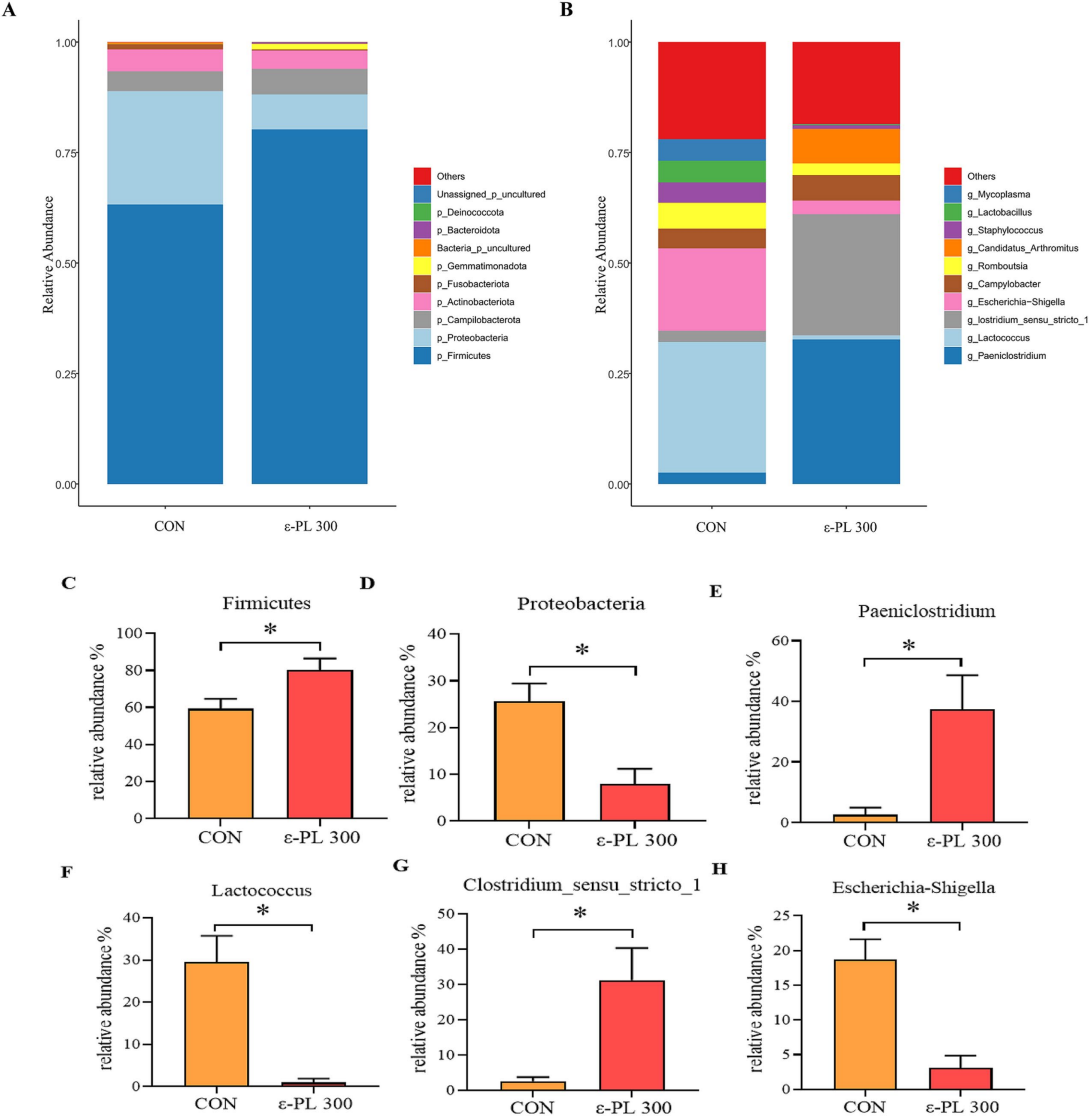
Effects of ε-PL supplementation on the relative abundance of bacterial taxa in ileal digesta of growing male minks (*n* = 8). **(A)** Top 10 phyla; **(B)** Top 10 genera; **(C,D)** the differential bacteria between the two groups at the phylum level; **(E–H)** the differential bacteria between the two groups at the genus level. CON, minks fed a basal diet; ε-PL 300, minks fed a basal diet supplemented with 300 mg/kg of ε-PL.* *p* < 0.05.

The top 10 bacterial phyla with the largest abundances in the control and 300 mg/kg ε-PL groups were Firmicutes, Proteobacteria, Campilobacterota, Actinobacteriota, Fusobacteriota, Gemmatimonadota, Bacteria_p_uncultured, Bacteroidota, Deinococcota and Unassigned_p_uncultured (Figure 2A). The top 10 bacterial genera in the control and 300 mg/kg *ε*-PL groups were *Paeniclostridium*, *Lactococcus*, *Clostridium_sensu_stricto_1*, *Escherichia-Shigella*, *Campylobacter*, *Romboutsia*, *Candidatus_Arthromitus*, *Staphylococcus*, *Lactobacillus*, and Mycoplasma (Figure 2B). Compared to the control, 300 mg/kg ε-PL significantly increased the relative abundance of Firmicutes, *Paeniclostridium* and *Clostridium_sensu_stricto_1* (*p* < 0.05) (Figures 2C,E,G) and reduced the relative abundance of Proteobacteria, *Lactobacillus*, and *Escherichia-Shigella* (*p* < 0.05) (Figures 2D,F,H).

#### 3.5.3 LEfSe analysis of intestinal microbiota

**Figure 3:**
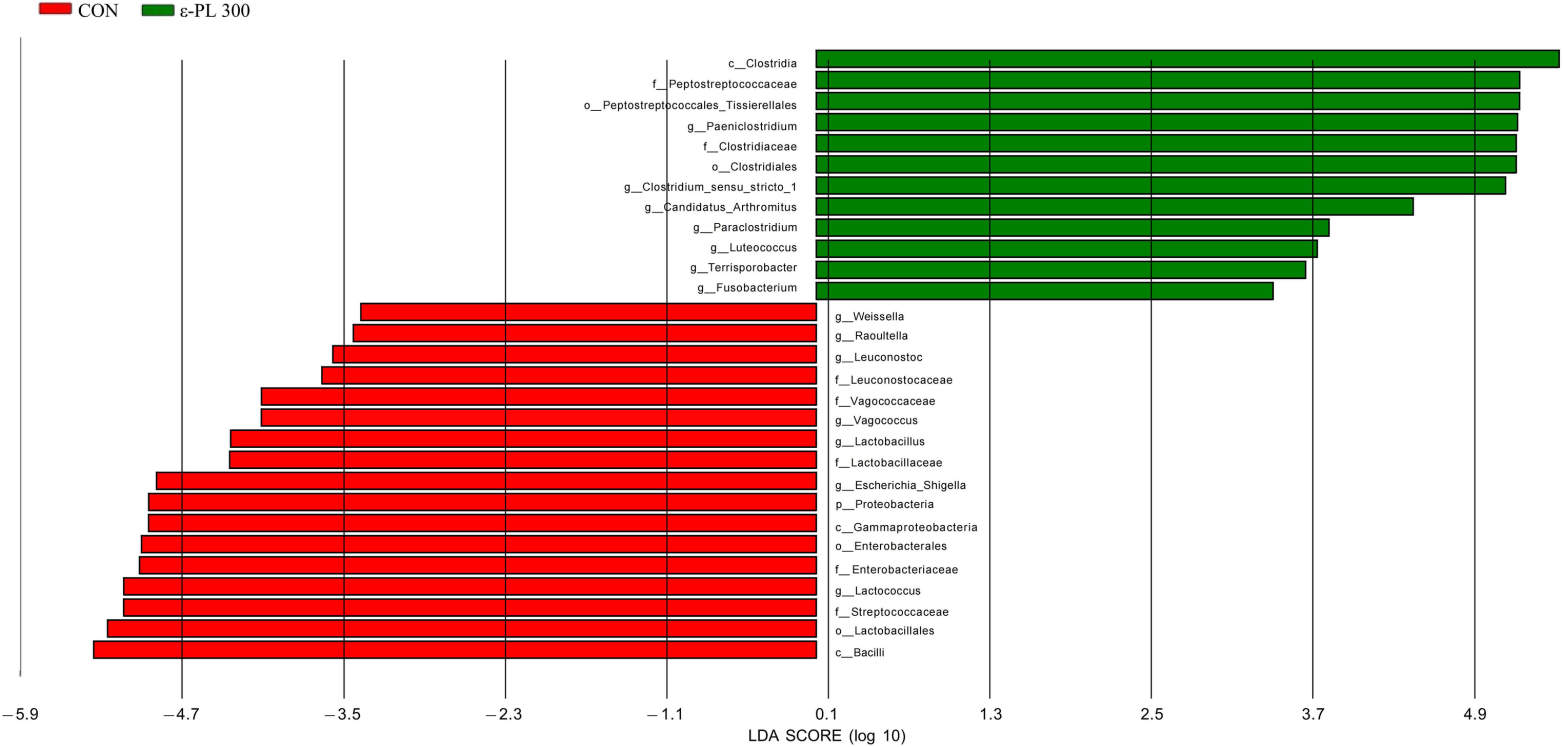
LEfSe analysis of the intestinal microbiota in growing male minks (n = 8). CON, minks fed a basal diet; ε-PL 300, minks fed a basal diet supplemented with 300 mg/kg of ε-PL.

LEfSe analysis showed that p_Proteobacteria, g_*Vagococcus*, f_Streptococcaceae, f_Enterobacteriaceae, g_*Escherichia_Shigella*, etc. in the control group exhibited higher LDA scores than those in the 300 mg/kg ε-PL group (Figure 3). Additionally, the bacteria enriched in the 300 mg/kg ε-PL group were f_Peptostreptococcaceae, g_*Candidatus_ Arthromitus*, g_*Clostridium_sensu_stricto_1*, g_*Luteococcus*, etc.

#### 3.5.4 Correlation analysis between the top 10 genera and health-related indicators

**Figure 4:**
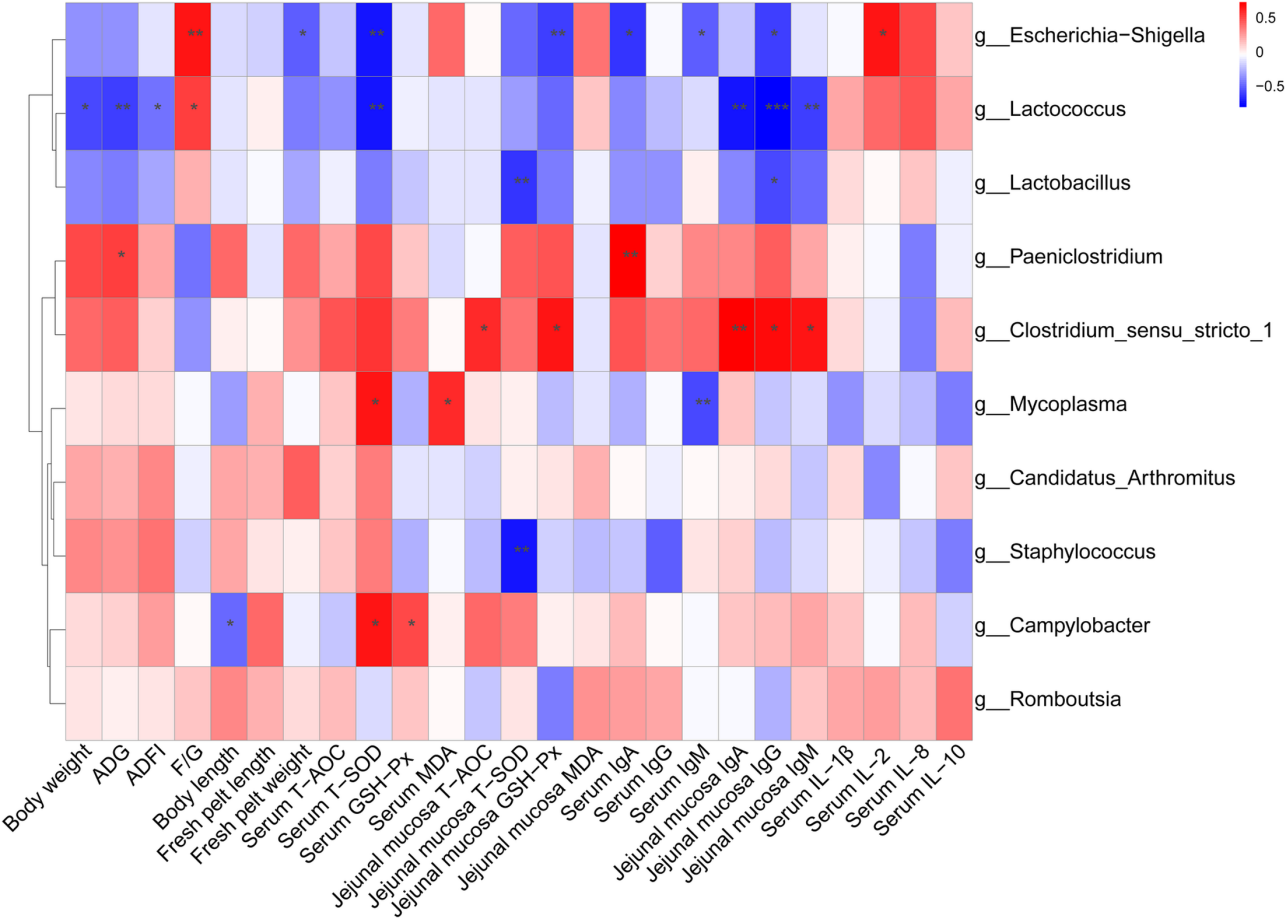
Spearman correlation analysis between the top 10 genera and health-related parameters. The color intensity indicates the strength of the association (red represents a positive correlation, while blue represents a negative correlation). *p < 0.05, **p < 0.01, ***p < 0.001.

As shown in Figure 4, the relative abundance of *Escherichia-Shigella* was positively correlated with the F/G ratio and serum IL-2 level, but was negatively correlated with fresh pelt weight, serum T-SOD activity, jejunal mucosal GSH-Px activity, serum IgA and IgM levels, as well as jejunal mucosal IgG level. The relative abundance of *Lactococcus* was positively correlated with the F/G ratio but negatively correlated with body weight, ADG, ADFI, serum T-SOD activity and jejunal mucosal IgA, IgM, and IgG levels. The relative abundance of *Lactobacillus* displayed a positive correlation with jejunal mucosal T-SOD activity and IgG level. The relative abundance of *Paeniclostridium* was positively correlated with ADG and serum IgA level. The relative abundance of *Clostridium_sensu_stricto_1* was positively correlated with jejunal mucosal T-AOC and GSH-Px activities, as well as IgA, IgM, and IgG levels. The relative abundance of *Mycoplasma* showed a positive correlation with serum T-SOD activity and MDA level, but a negative correlation with serum IgM level. The relative abundance of *Staphylococcus* was negatively correlated with jejunal mucosal T-SOD activity. The relative abundance of *Campylobacter* was positively correlated with jejunal mucosal T-SOD and GSH-Px activities but negatively correlated with body length.

## 4 Discussion

The growth stage is a critical period for minks, as their peak growth rate and daily weight gain directly determine adult body size. In this study, dietary supplementation with 300, 400 and 500 mg/kg *ε*-PL significantly increased ADG and final body weight and decreased F/G during the entire 8 weeks. ε-PL has a broad-spectrum inhibitory effect on the growth and reproduction of Gram-positive and Gram-negative bacteria and molds (Li et al., 2014). ε-PL can inhibit the deterioration of wet feed in hot environments, which may explain the observed improvements in daily weight gain and feed intake in minks. In addition, ε-PL can be digested into lysine, a growth-promoting amino acid, which participates directly in protein synthesis and significant influences growth and development (Kiess et al., 2013). Similar to our results, Wang et al. (2022) reported that the dietary supplementation with antimicrobial peptides Gal-13 significantly increased ADG during the 7 to 42 days of age and final body weight at day 42 of age in broiler chickens. Zhang et al. (2021) showed that dietary supplementation with antimicrobial peptide plectasin remarkably increased ADG and ADFI, and reduced F/G in yellow-feathered chickens during days 1 to 63 of age. Yoon et al. (2014) found that supplementation with antimicrobial peptides A3 and P5 in diets increased ADG and decreased F/G in weaned piglets during days 0 to 28 of age.

There is a close correlation between the antioxidant systems of the body and health, and excess free radicals can cause damage to tissues and cells, leading to an unstable internal environment (Singh et al., 2019). Various antioxidant molecules and enzymes affect the overall antioxidant activity of the organism, and T-AOC, T-SOD, and GSH-Px are important indicators of the organism’s ability to scavenge free radicals (Zhang et al., 2020b). Highly active SOD reduces free radical-induced damage and protects cells from oxidative stress, thus maintaining normal cell function and health status. GSH-Px can reduce endogenous hydrogen peroxide and hydroxyl radicals, thereby reducing the levels of lipid peroxides and free radicals in the blood (Yoon et al., 2012). As a secondary metabolite of lipid oxidation, MDA is often used as an indicator of lipid peroxidation (Wu et al., 2020). Deng et al. (2020) found that *ε*-PL can effectively scavenge free radicals and has robust antioxidant activity in *in vitro* tests. Liu et al. (2022) showed that dietary supplementation of immobilized antimicrobial peptides increased serum T-SOD and GSH-Px activities in weaned piglets. In this study, dietary supplementation with 300 mg/kg ε-PL significantly increased serum T-SOD activity and jejunal mucosal T-SOD and GSH-Px activities, indicating that *ε*-PL could effectively improve antioxidant function and reduce oxidative damage in growing minks.

Animal-sourced feeds, such as fish and offal, constitute the major part of mink diets, however, they are prone to harbor bacteria in high-temperature environments, which challenges the immune system of minks. Under antigenic stimulation, the immune systeminduces B lymphocytes to proliferate and differentiate into plasma cells, which produce immunoglobulins that are widely distributed in serum and tissue fluids (Ehrenstein and Notley, 2010). IgG is the main immunoglobulin involved in the humoral immune responses, with the ability to activate complement, neutralizing toxins, and regulating phagocytosis (Ulfman et al., 2018). IgM is the earliest immunoglobulin generated by the immune system during infections, playing a crucial role in innate immunity (Keyt et al., 2020). IgA functions primarily in the mucosal defense system, such as in the respiratory and digestive systems, and prevents pathogenic microorganisms from adhering to mucosal membranes (Gao et al., 2023). Liu et al. (2022) demonstrated that adding immobilized antimicrobial peptides to the diet markedly elevated serum concentrations of IgG and IgM in weaned piglets. Yuan et al. (2015) observed that dietary supplementation with antimicrobial peptides increased the IgG, IgM and IgA levels in weaned piglets. In this study, 300 and 400 mg/kg *ε*-PL increased serum IgA and IgM levels, and 200, 300 and 400 mg/kg *ε*-PL increased mucosal IgA and IgG levels, indicating that dietary ε-PL supplementation promotes the immune response in growing minks.

Inflammatory cytokines are secreted by immune cells involved in inflammatory responses, and they are mainly classified into pro-inflammatory cytokines and anti-inflammatory cytokines (Chauhan et al., 2021). IL-2 is a pro-inflammatory cytokine that can promote T cell growth, enhance the activity of natural killer cells, and induce the production of cytotoxic T lymphocytes (Lokau et al., 2023; Liao et al., 2021). As a chemokine, IL-8 mainly attracts and activates neutrophils, leading to local inflammatory responses in the body, thus exerting immune function (Baggiolini and Clark-Lewis, 1992). IL-1β is also a pro-inflammatory cytokine, which initiates inflammatory responses and promotes the clearance of pathogens during infections and injuries (Cheng et al., 2019). IL-10 is an anti-inflammatory cytokine, which can inhibit inflammatory Th cells and immunopathological processes, thereby maintaining tissue homeostasis (Fang and Zhu, 2019). Feng et al. (2020) found that intraperitoneal injection with antimicrobial peptide cathelicidin-BF reduced the diarrhea index and serum levels of IL-6 and IL-8 in piglets with post-weaning diarrhea. In addition, dietary supplementation with antimicrobial peptide Gal-13 significantly decreased the expression of IL-2 in the spleen of piglets (Wang et al., 2022). In this study, the results showed that *ε*-PL supplementation markedly reduced serum IL-2 and IL-8 levels, indicating that dietary supplementation with ε-PL could effectively reduce serum pro-inflammatory cytokine levels, thus alleviating the inflammatory responses in growing minks.

The gut microbiota is crucial for intestinal homeostasis and has an important impact on the host’s nutrient absorption and immune system (Ignacio et al., 2016). Alpha diversity is used to assess the richness and diversity of microbial communities in the gut. In this study, dietary supplementation with ε-PL had no significant effect on the alpha diversity of the gut microbiota in growing minks, which is similar to the results reported in laying hens (Wang et al., 2024). Beta diversity measures the differences in microbial communities between samples. In this study, PCoA and NMDS analyses showed an obvious separation in beta diversity between the control and 300 mg/kg *ε*-PL groups, suggesting that dietary ε-PL supplementation could alter the structure of the ileal microbiota.

In this study, 300 mg/kg ε-PL increased the relative abundance of Firmicutes and *Clostridium_sensu_stricto_1* and decreased the relative abundance of Proteobacteria and *Escherichia-Shigella.* Firmicutes are one of the most abundant bacterial phyla in the gut, which promotes cellulose catabolism and glucose metabolism to provide energy for the body (Xue et al., 2016). As members of Firmicutes, *Clostridium* spp. exert the probiotic effects by producing butyric acid, strengthening the intestinal barrier and regulating intestinal immunity (Guo et al., 2020). Shin et al. (2015) showed that proteobacteria include various pathogens, such as *Vibrio cholerae*, *Salmonella*, *Escherichia coli*, and *Helicobacter pylori*, and they are usually benign at low abundances but may become pathogenic when the intestinal environment changes dramatically. *Escherichia-Shigella* can colonize the intestine and secrete toxins, causing metabolic disorders in the intestinal epithelial cells, which damage the intestinal mucosal architecture and contributes to immune dysfunction in the gut (Dubreuil et al., 2016). In this study, LEfSe analysis showed that Proteobacteria and *Escherichia-Shigella* were enriched in the intestinal tracts of control minks, while *Candidatus_Arthromitus* was enriched in minks fed 300 mg/kg *ε*-PL. *Candidatus_Arthromitus* can regulate T cell differentiation and stimulate IgA secretion, enhancing resistance to foreign pathogenic bacteria (Ohashi et al., 2010), which may explain why dietary ε-PL supplementation increased immunoglobulin levels and enhanced immunity in growing minks. Zhang et al. (2020a) reported that dietary ε-PL supplementation remarkably increased the abundance of the phylum *Firmicutes* in the ileum of Ningxiang pigs. Cao et al. (2024) reported that dietary supplementation with antimicrobial peptide microcin J25 dramatically increased the relative abundance of Firmicutes and reduced the relative abundance of Proteobacteria and Enterobacteriaceae in pigeon squabs. In addition, dietary supplementation of antimicrobial peptide Microcin C7 reduced the number of *Escherichia coli* in the cecum of broiler chickens (Dai et al., 2022). Therefore, our results suggest that dietary *ε*-PL supplementation may improve the growth of potential beneficial bacteria and inhibit the growth of potential pathogenic bacteria in growing minks.

The results of Spearman correlation analysis showed that the relative abundance of *Escherichia-Shigella* was positively correlated with the F/G ratio and negatively correlated with the fresh pelt weight. Similarly, a negative correlation between *Escherichia-Shigella* and growth performance was also observed in a study of ammonia exposure in broiler chickens (Han et al., 2021). In this study, the improved growth performance in minks of the 300 mg/kg ε-PL group may be attributed to the lower abundance of *Escherichia-Shigella* compared with the control group. Our results showed that the relative abundance of *Clostridium_sensu_stricto_1* was positively correlated with the jejunal mucosal antioxidant indicators and immunoglobulin levels, which indicated that the enhanced antioxidant and immune function of ε-PL may be associated with the increased relative abundance of *Clostridium_sensu_stricto_1*. Consistent with the results of this study, some feed additives, such as zinc oxide (Xiao et al., 2023) and *Enterococcus faecium* (Zhang et al., 2025), have also been found to increase the relative abundance of *Clostridium_sensu_stricto_1* after administration. Generally, the top 10 genera in the ileum have been found to be mainly associated with growth performance, antioxidant enzyme activities and immunoglobulin levels, which further confirms that microorganisms can affect the health of the body.

## 5 Conclusion

Dietary supplementation of *ε*-PL enhanced growth performance, boosted antioxidant capacity, and strengthened immune functions, and improved the composition of intestinal microbiota in male minks during the breeding period. A dose of 300 mg/kg ε-PL in the diet was recommended for male minks.

## Data Availability

The sequencing data of gut microbial diversity are deposited in the China National GeneBank DataBase under the accession number CNP0006834.
